# Impact of myocardial scars on left ventricular deformation in type 2 diabetes mellitus after myocardial infarction by contrast-enhanced cardiac magnetic resonance

**DOI:** 10.1186/s12933-021-01407-2

**Published:** 2021-10-25

**Authors:** Yue Gao, Hua-yan Xu, Ying-kun Guo, Xiao-ling Wen, Rui Shi, Yuan Li, Zhi-gang Yang

**Affiliations:** 1 Department of Radiology, West China Hospital, Sichuan University, 37# Guo Xue Xiang, Chengdu, 610041 Sichuan China; 2 Department of Radiology, Key Laboratory of Birth Defects and Related Diseases of Women and Children of Ministry of Education, West China Second University Hospital, Sichuan University, Chengdu, China; 3 Department of Radiology, West China Fourth Hospital, Sichuan University, 18# Section 3, Renmin South Road, Chengdu, Sichuan Province China

**Keywords:** Diabetic cardiomyopathy, Myocardial infarction, Myocardial scar, Cardiac magnetic resonance

## Abstract

**Background:**

Type 2 diabetes mellitus (T2DM) is a major risk factor for coronary artery disease and myocardial infarction (MI). The interaction of diabetic cardiomyopathy and MI scars on myocardial deformation in T2DM patients is unclear. Therefore, we aimed to evaluate myocardial deformation using cardiac magnetic resonance (CMR) in T2DM patients with previous MI and investigated the influence of myocardial scar on left ventricular (LV) deformation.

**Methods:**

Overall, 202 T2DM patients, including 46 with MI (T2DM(MI+)) and 156 without MI (T2DM(MI−)), and 59 normal controls who underwent CMR scans were included. Myocardial scars were assessed by late gadolinium enhancement. LV function and deformation, including LV global function index, LV global peak strain (PS), peak systolic strain rate (PSSR), and peak diastolic strain rate (PDSR), were compared among these groups. Correlation and multivariate linear regression analyses were used to investigate the relationship between myocardial scars and LV deformation.

**Results:**

Decreases were observed in LV function and LV global PS, PSSR, and PDSR in the T2DM(MI+) group compared with those of the other groups. Reduced LV deformation (*p* < 0.017) was observed in the T2DM(MI+) group with anterior wall infarction. The increased total LV infarct extent and infarct mass of LV were related to decreased LV global PS (radial, circumferential, and longitudinal directions; *p* < 0.01) and LV global PSSR (radial and circumferential directions, *p* < 0.02). Multivariate analysis demonstrated that NYHA functional class and total LV infarct extent were independently associated with LV global radial PS (β =  − 0.400 and β =  − 0.446, respectively, all *p* < 0.01; model R^2^ = 0.37) and circumferential PS (β = 0.339 and β = 0.530, respectively, all *p* < 0.01; model R^2^ = 0.41), LV anterior wall infarction was independently associated with LV global longitudinal PS (β = 0.398, *p* = 0.006).

**Conclusions:**

The myocardial scarring size in T2DM patients after MI is negatively correlated with LV global PS and PSSR, particularly in the circumferential direction. Additionally, different MI regions have different effects on the reduction of LV deformation, and relevant clinical evaluations should be strengthened.

## Introduction

Diabetic cardiomyopathy (DCM) is defined as myocardial dysfunction independent of coronary artery disease and hypertension that can lead to heart failure [1, 2]. Diastolic dysfunction is one of the important indicators of early left ventricular (LV) dysfunction before reduced LV ejection fraction in DCM, and the impaired global longitudinal strain was associated with cardiovascular events in type 2 diabetes mellitus (T2DM) patients [3–5]. Myocardial microvascular dysfunction, and remodeling of the extracellular matrix are related to contractile dysfunction in DCM [6]. Coronary artery disease and myocardial infarction (MI) are major causes of global morbidity and mortality, and T2DM is considered a major risk factor for coronary artery disease, T2DM patients are at a high risk of MI and have a poor prognosis [7, 8]. Previous studies have pointed out that the MI size and transmural type in MI patients have an important effect on prognosis and survival. In patients with previous MI, myocardial scarring causes weakened or even contradictory LV wall movement, fibrotic repair of the infarcted area, and compensatory cardiomyocyte hypertrophy in remote infarcted areas, all of which affect LV myocardial deformation [9]. Currently, few studies have investigated the effects of myocardial scar on myocardial deformation after MI in T2DM patients with DCM.

Cardiac magnetic resonance (CMR) imaging, which has been widely used in the last decades in clinical practice, provides information on various characteristics of cardiac structure, function, and myocardial tissue [10–12]. CMR tissue tracking has been used to measure myocardial deformation [13]. The late gadolinium enhancement (LGE) sequence detects and assesses the myocardial scar with high spatial resolution and quantifies the MI area [14]. Therefore, this study aimed to evaluate LV deformation using CMR in T2DM patients with previous MI and to investigate the influence of MI scar on LV deformation.

## Methods and materials

### Study population

**Fig. 1:**
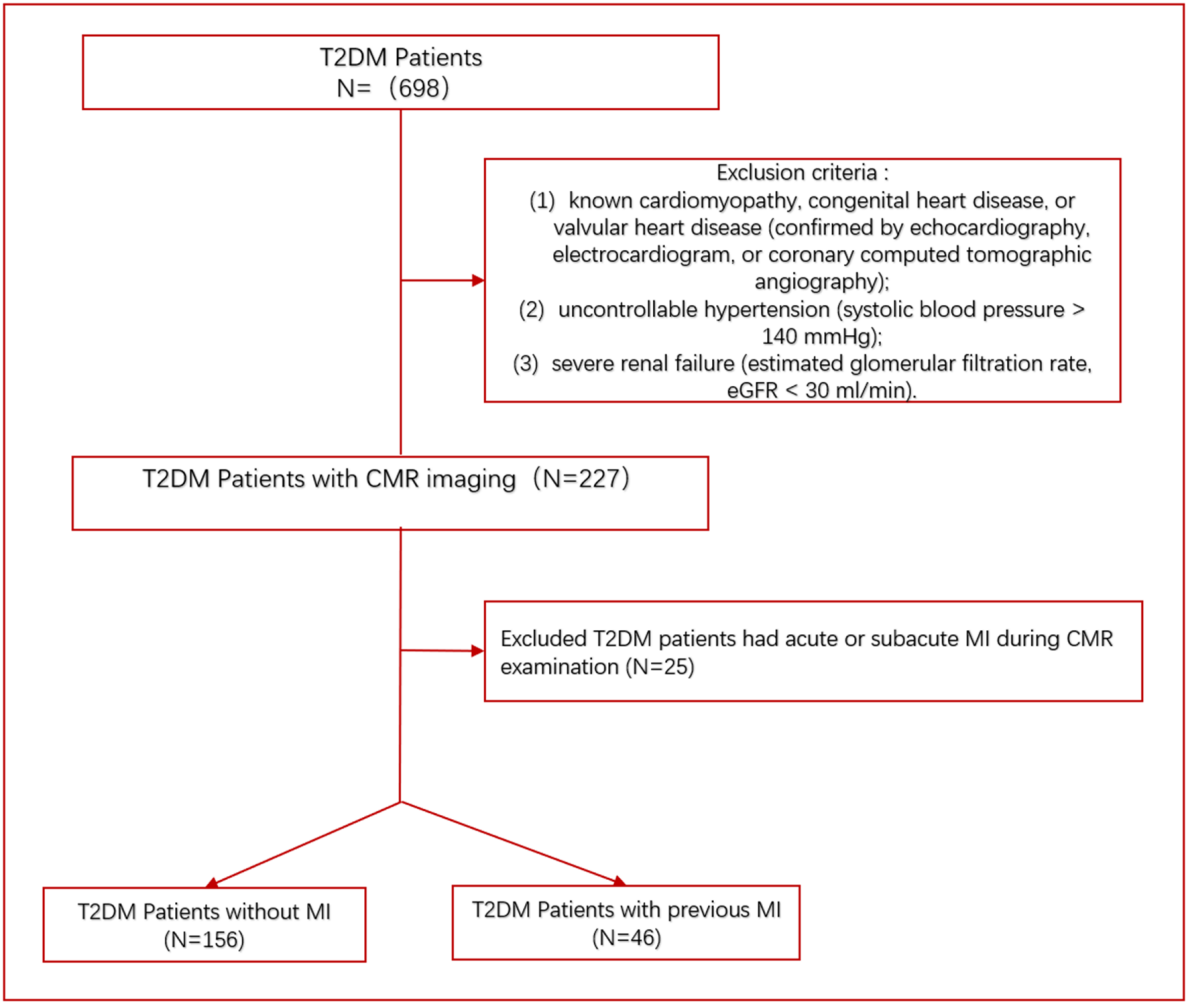
Flow chart of the study

The study protocol was approved by Biomedical Research Ethics Committee of our hospital. Initially, we retrospectively enrolled 698 patients diagnosed with T2DM according to the World Health Organization standards between January 2015 and May 2021, and had completed CMR examinations [15]. The exclusion criteria were as follows: (1) known cardiomyopathy, congenital heart disease, or valvular heart disease (confirmed by echocardiography, electrocardiogram, or coronary computed tomographic angiography); (2) uncontrollable hypertension (systolic blood pressure > 140 mmHg); (3) severe renal failure (estimated glomerular filtration rate, eGFR < 30 mL/min); and (4) acute or subacute MI patients. Acute MI patients might have edema myocardium and stunned myocardium, which affect the measurement of CMR parameters. Following these criteria, a total of 227 T2DM patients were included in this study. Of these, 156 T2DM patients (48 males and 106 females; mean age 55.55 ± 11.8 years) had no previous MI or coronary revascularization, 25 T2DM patients had acute or subacute MI during CMR examination, and 46 TDM patients (28 males and 18 females; mean age 61.54 ± 9.11 years) had previous acute MI (> 6 months). Finally, we excluded the 25 T2DM patients who had acute or subacute MI. This study defined MI according to the European Society of Cardiology/American College of Cardiology/American Heart Association committee criteria [16]. In addition, age-, sex-, and body mass index-matched healthy volunteers were recruited in the control group. Exclusion criteria for the control group were as follows: (1) DM or impaired glucose tolerance; (2) known acute or chronic diseases such as hypertension; (3) presence of dyspnea, chest pain, palpitation, or other cardiovascular disease-related symptoms; (4) electrocardiogram abnormalities; and (5) CMR detected abnormalities (perfusion defect, local or diffuse myocardial late-gadolinium enhancement, abnormal ventricular motion, valvular stenosis, etc.). Finally, a total of 59 healthy controls (27 males and 32 females; mean age 58.53 ± 8.23 years) were included in this study. A detailed flow chart of the present study is presented in Fig. 1.

Clinical characteristics, family history, surgery history, medication, and serum biochemical indexes of all patients and healthy controls were collected. Blood sampling for serum biochemical indexes was performed within one week of the CMR scan without changing the subject's medication regimen.

### CMR scanning protocol

All subjects underwent CMR scan using a 3.0-T whole-body scanner (Skrya; Siemens Medical Solutions, Erlangen, Germany) in the supine position. A standard ECG-triggering device was used, and end-inspiratory breath-holding was performed. Following a survey scan, cine images such as long-axis four-chamber views and short-axis two-chamber views were acquired using a steady-state free-precession sequence (temporal time = 39.34 ms, echo time = 1.22 ms, flip angle = 40°, slice thickness = 8 mm, field of view = 360 × 300 mm^2^, matrix size = 256 × 166). Gadobenate dimeglumine (MultiHance; Bracco, Milan, Italy) was intravenously injected at a dose of 0.2 ml/kg body weight at an injection rate of 2.5–3.0 mL/s, followed by a 20 mL saline flush at a rate of 3.0 mL/s. LGE images were acquired in the corresponding slice position as the cine imaging 10–15 min after contrast injection. The images were obtained using a phase-sensitive inversion recovery sequence with the following parameters: temporal time = 300 ms, echo time = 1.44 ms, flip angle = 40°, slice thickness = 8 mm, field of view = 275 × 400 mm^2^, matrix size = 256 × 184.

### CMR data analysis

**Fig. 2:**
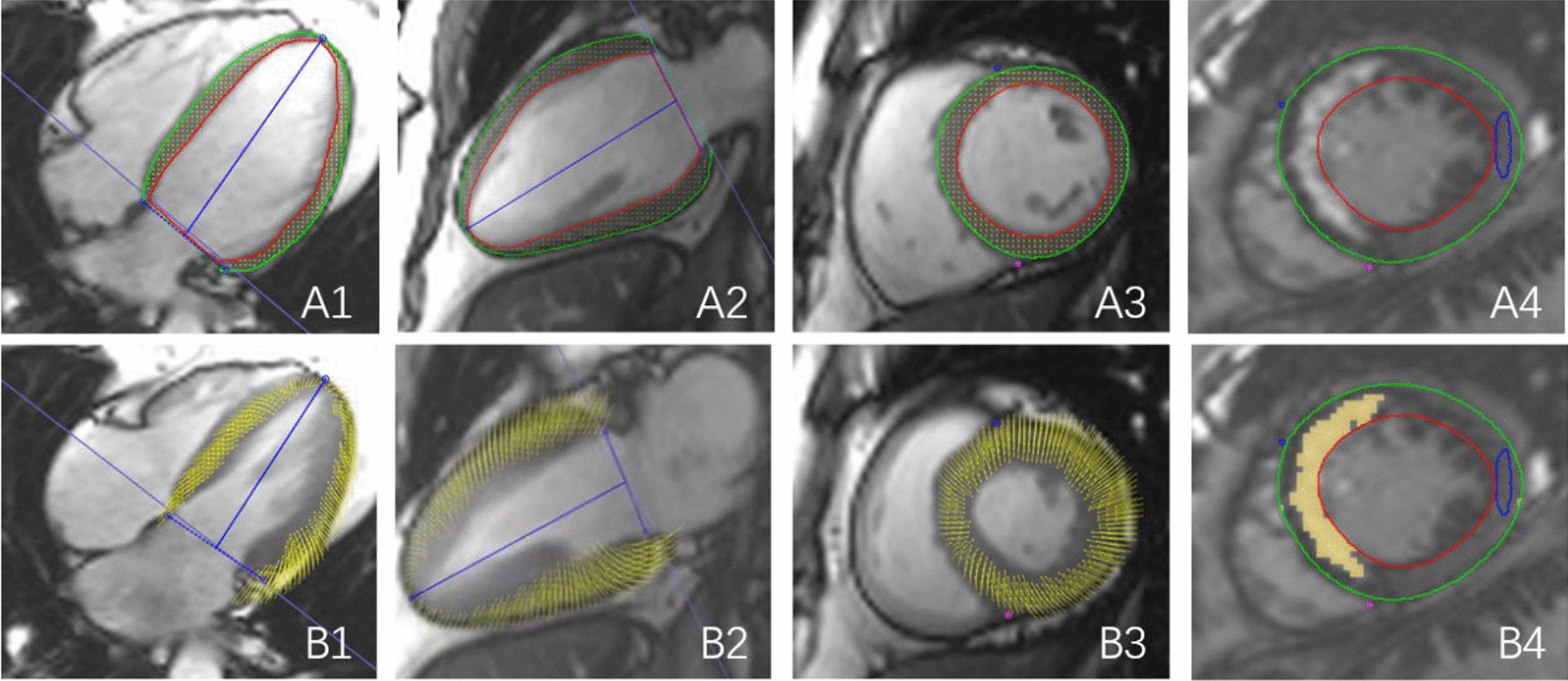
Measurement of LV global strain and enhanced area in LGE. Cardiac magnetic resonance tissue tracking in short-axis and long-axis two-chamber and four-chamber cine images at end-diastole (**A1**–**3**) and end-systole (**B1**–**3**). LGE images for quantification of infarct size (**A4**, **B4**) the signal intensity of yellow region was five standard deviations above the mean intensity of the normal myocardium (blue circle)

All CMR data were uploaded to an offline workstation using a semi-automated software (Cvi42; Circle Cardiovascular Imaging, Inc., Calgary, Canada). Endocardial and epicardial traces were manually delineated by two experienced radiologists in the serial short-axis slices during the end-diastolic and end-systolic phases (Fig. 2). Papillary muscles were considered as part of the ventricular cavity, and epicardial fat was excluded. Subsequently, LV functional parameters and LV mass were automatically determined. LV remodeling was characterized by the ratio of LV mass to LV end-diastolic volume (LVEDV) (LVMVR). The LV global function index (LVGFI) was calculated using the following formula:

$${\text{LVGFI}} = \left\{ {{\text{LVSV}}/\left[ {\left( {{\text{LVEDV}} + {\text{LVESV}}} \right)/{2} + \left( {{\text{LV mass}}/{1}.0{5}} \right)} \right]} \right\} \times {1}00.$$

For LV myocardial strain analysis, long-axis 2-chamber, 4-chamber, and short-axis slices were loaded into the 3-dimensional tissue tracking module. The LV global myocardial strain parameters, including radial, circumferential, and longitudinal peak strain (PS); peak systolic strain rate (PSSR); and peak diastolic strain rate (PDSR) were acquired automatically. For LGE imaging analysis, LV segment–based analysis was performed in accordance with the 16-segment model of the American Heart Association. The hyper-enhanced myocardium area was defined as the myocardial scar on the LGE short-axis images when the signal intensity was five standard deviations above the mean intensity of the normal myocardium (Fig. 2A4, B4) [17]. We assessed the extent of the LGE regions involving the LV wall, by dividing it into the interventricular septum, anterior wall, inferior wall, and lateral wall using the 16-segment model. Two radiologists evaluated the images separately, and if the results were inconsistent, they discussed and agreed on the result.

### Variability analysis

To determine intra-observer variability, LV deformation and LGE parameters in 70 random cases that included 50 T2DM patients and 20 normal controls were measured twice within a week by a radiologist. Next, a second radiologist, who was blinded to the findings of the first investigator reperformed the measurements to assess the interobserver variability.

### Statistical analysis

Statistical analyses were performed with SPSS (version 21.0 for Windows; SPSS, Inc., Chicago, IL, USA). Data were expressed in the format of mean ± standard deviations or median interquartile range for continuous variables and frequencies for categorical variables. One-way analysis of variance test was performed to evaluate the differences among the following groups: T2DM(MI+), T2DM(MI−) and normal control. Based on Bonferroni’s correction for multigroup comparisons, p-values of < 0.017 were considered statistically significant. Spearman’s and Pearson’s correlation analyses were conducted to identify the relationship between infarction and cardiac deformation. Pearson’s correlation was used between continuous variables and Spearman’s correlation was used to analyze the rank correlation Moreover, multivariable stepwise linear regression analysis was employed to identify the relationship between infarction parameters and cardiac dysfunction. Variables with a p-value of 0.1 in the univariable analyses were then included in a stepwise multivariable analysis based on a linear regression model. A p-value of < 0.05 was considered statistically significant.

## Results

### Patient characteristics

Of the 202 T2DM patients, 156 were in the T2DM(MI−) group (48[30.7%]) males, mean age 55.55 ± 11.80 years) and 46 in the T2DM(MI+) group (28[60.8%]) males, mean age 61.54 ± 9.11 years). Table 1 presents the patients’ baseline characteristics, cardiovascular risk factors, metabolic parameters, and medications. The results showed no statistically significant differences in the baseline characteristics among these groups, except for a higher number of males in the T2DM(MI+) group than in the T2DM(MI−) group. Regarding cardiovascular risk factors, more patients were previous or current smokers (50.5% vs. 28.3%) in the T2DM(MI+) group than that in the T2DM(MI−) group, there was no difference in hyperlipidemia or family history of T2DM between the groups. HbA1c and fasting plasma glucose showed no statistically significant differences between the T2DM(MI+) and T2DM(MI−) groups.

**Table 1:** Baseline characteristics of the study cohort

	Normal (n = 59)	T2DM (MI−) (n = 156)	T2DM (MI+) (n = 46)
Baseline characteristics			
Age, years	58.53 ± 8.23	55.55 ± 11.8	61.54 ± 9.11
Male, n (%)	27 (48.2%)	48 (30.7%)	28 (60.8%)^§^
BMI, kg/m^2^	23.17 ± 3.14	23.67 ± 3.06	25.32 ± 3.35
Systolic blood pressure, mmHg	120.19 ± 8.85	127.96 ± 13.11	123.67 ± 19.27
Diastolic blood pressure, mmHg	75.73 ± 10.36	79.88 ± 9.88	77.89 ± 12.76
Heart rate, bpm	73.28 ± 10.85	72.67 ± 15.42	73.80 ± 14.60
Cardiovascular risk factors			
Previous/current smoker, n (%)	9 (15.2%)	44 (28.3%)	23 (50.5%)^§^
Hyperlipidemia	0	29 (18.5%)	8 (17.4%)
Family history of DM	0	32 (20.5%)	11 (23.9%)
Pervious PCI, n (%)	0	0	20 (43.5) %
Previous CABG, n (%)	0	0	1 (2.2%)
NYHA functional class, n			
**I**	–	–	3 (6.5%)
**II**	–	–	27 (58.7%)
**III**	–	–	15 (32.6%)
**IV**	–	–	1 (2.2%)
Culprit vessel n (%)			
LM	–	–	0
LAD	–	–	14 (30.43%)
LCx	–	–	6 (13.0%)
RCA	–	–	14 (30.43%)
Metabolic characteristics			
HbA1c, %	5.46 ± 0.34	7.82 ± 0.87	8.10 ± 1.32
Fasting plasma glucose, mmol/L	5.08 ± 0.45	8.28 ± 1.23	8.78 ± 2.34
eGFR, mL/min/1.73 m^2^	107.23 ± 8.45	95.84 ± 9.32	79.60 ± 10.45
NT-proBNP	–	–	874 (204.75–2032.75)
Medication, n (%)			
Aspirin, n (%)	–	17 (6.5%)	28 (60.8%)
ß-blockers, n (%)	–	0	11 (19.6%)
ACEI/ARB, n (%)	–	5 (3.2%)	16 (34.7%)
Diuretics	–	1 (0.06%)	13 (28.2%)
Calcium-channel blocker	–	8 (5.1%)	5 (10.8%)
Insulin	–	36 (23.1%)	7 (15.2%)
Statin, n (%)	–	6 (3.8%)	14 (30.4%)

*T2DM* type 2 diabetes mellitus, *MI* myocardial infarct, *BMI* body mass index, *PCI* percutaneous transluminal coronary intervention, *CAGB* coronary artery bypass grafting, *NYHA* New York Heart Association, *HbA1c* glycated hemoglobin, *eGFR* estimated glomerular filtration rate, *ACEI* angiotensin converting enzyme inhibitor。

^§^p < 0.017 versus T2DM patients without MI (Bonferroni’s)

In the T2DM(MI+) group, 20 patients received the percutaneous coronary intervention, and one patient received coronary artery bypass grafting surgery. Thirty-four patients were identified with culprit vessels, of which 14 (30.43%) were the left anterior descending coronary artery (LAD), 6 (13.0%) were the left circumflex coronary artery (LCx), and 14 (30.43%) were the right coronary artery (RCA).

### Comparison of LV function and deformation among three groups

The CMR imaging results for LV function and deformation are summarized in Table 2. LVESVi (LVESV index) was higher in the T2DM(MI−) group than in the control group. LVESVi, and LV mass were higher (all p < 0.005) in the T2DM(MI+) group compared with the T2DM(MI−) and normal control groups. Meanwhile, LVSVi (T2DM(MI+) vs. T2DM(MI−): 39.33 (31.96–46.71) vs. 48.17 (41.04–54.36); T2DM(MI+) vs. control: 39.33 (31.96–46.71) vs. 47.52 (42.13–53.67), p < 0.001) and LVGFI (T2DM(MI+) vs. T2DM(MI−): 27.37 (18.12–40.02) vs. 48.99 (44.38–54.33); T2DM(MI+) vs. control: 27.37 (18.12–40.02) vs. 51.56 (48.03–56.06)), p < 0.001) were lower in the T2DM(MI+) group than in the T2DM(MI−) and control groups.

**Table 2:** CMR findings between normal individuals, T2DM (MI−) group and T2DM (MI+) group

	Normal (n = 59)	T2DM (MI (−)) (n = 156)	T2DM (MI (+)) (n = 46)
LVEDVi, mL/m^2^	70.75 (63.97–80.18)	76.49 (67.45–84.18)	114.56 (80.18–137.75)*
LVESVi, mL/m^2^	23.64 (19.16–28.39)	26.46 (20.92–32.32)*	72.52 (39.62–102,18)*^§^
LVSVi, mL/m^2^	47.52 (42.13–53.67)	48.17 (41.04–54.36)	39.33 (31.96–46.71)*^§^
LVCO, L/min	5.54 (5.00–6,63)	5.85 (4.56–7.00)	5.11 (3.95–6.25)
LVEF, %	66.14 (62.85–70.86))	64.74 (59.32–69.69)	38.26 (24.75–55.68)*^§^
LV mass, g/m^2^	77.61 (68.40–88.32)	91.86.86 (74.13–111.40)	127.80 (103.68,156.91) *^§^
LVGFI, %	51.56 (48.03–56.06)	48.99 (44.38–54.33)	27.37 (18.12–40.02)^§^
PS, %			
Radial	35.32 (32.38–40.72)	32.11 (25.73–38.83)	14.71 (9.29–22.83)*^§^
Circumferential	− 20.92 (− 22.67 to (− 18.84))	− 19.56 (− 21.47 to (− 17.64))	− 11.11 (− 16.10 to (− 8.68))*^§^
Longitudinal	− 15.03 (− 16.68 to (− 12.31))	− 12.24 (− 14.67 to (− 10.08))*	− 7.41 (− 9.95 to (− 4.75)) *^§^
PSSR, 1/s			
Radial	2.05 (1.77–2.48)	1.745 (1.41–2.26)	0.83 (0.61–1.44)*^§^
Circumferential	− 1.02 (− 1.15 to (− 0.92))	− 0.98 (−1.13 to (− 0.86))	− 0.61 (− 0.81 to (− 0.45))*^§^
Longitudinal	− 0.75 (− 0.87 to (− 0.67))	− 0.69 (− 0.86 to (− 0.55))	− 0.38 (− 0.56 to (0.20))*^§^
PDSR, 1/s			
Radial	− 2.80 (− 3.22 to (− 2.12))	− 2.05 (− 2.65 to (− 1.58))	− 0.83 (− 1.24 to (− 0.51))*^§^
Circumferential	1.32 (1.19–1.53)	1.12 (0.97–1.36)*	0.65 (0.50–0.93)*^§^
Longitudinal	0.93 (0.70–1.11)	0.79 (0.60–0.97)*	0.49 (0.33–0.62)*
Infarct size, g	–	–	20.69 (11.69–32.12)
Infarct size, g % of LV	–	–	17.96 (11.07–25.26)
Total LV infarct extent (%)	–	–	17.12 (9.58–27.53)
Infarct territory, n (%)		–	
Interventricular septum	–	–	22 (47.8%)
Inferior	–	–	20 (43.4%)
Lateral	–	–	13 (28.2%)
Anterior	–	–	12 (26.09%)

Data are presented as median (25th, 75th percentile)

*LVEDVi* left ventricular end diastolic volume index, *LVESVi* left ventricular end systolic volume index, *LVSVi* left ventricular stroke volume index, *LVEF* left ventricular ejection fraction, *LVGFI* left ventricular global function index, *MI* myocardial infarction, *PDSR* peak diastolic strain rate, *PS* peak strain, *PSSR* peak systolic strain rate

^*^p < 0.017 versus normal group (Bonferroni’s)

^§^p < 0.017 versus T2DM patients without MI (Bonferroni’s)

Regarding LV deformation, the global radial, circumferential, and longitudinal PS (all *p* < 0.001) were lower in the T2DM(MI+) group than in the T2DM(MI−) and control groups. The global longitudinal PS (*p* < 0.017) was lower in the T2DM(MI−) group than in the control group. There was no statistically significant difference in global radial PS between the T2DM(MI−) and control group. The global radial, circumferential and longitudinal PSSR and PDSR (all *p* < 0.001) were significantly lower in the T2DM(MI+) group than in the T2DM(MI−) and control groups. Except for longitudinal PDSR in the T2DM(MI+) group compared with the T2DM(MI−) group. For the T2DM(MI−) group, the circumferential and longitudinal PDSR were lower than those in the control group (*p* < 0.017).

### LV infarct characteristics analysis

#### Association between LV deformation and LGE size in T2DM patients with MI

**Fig. 3:**
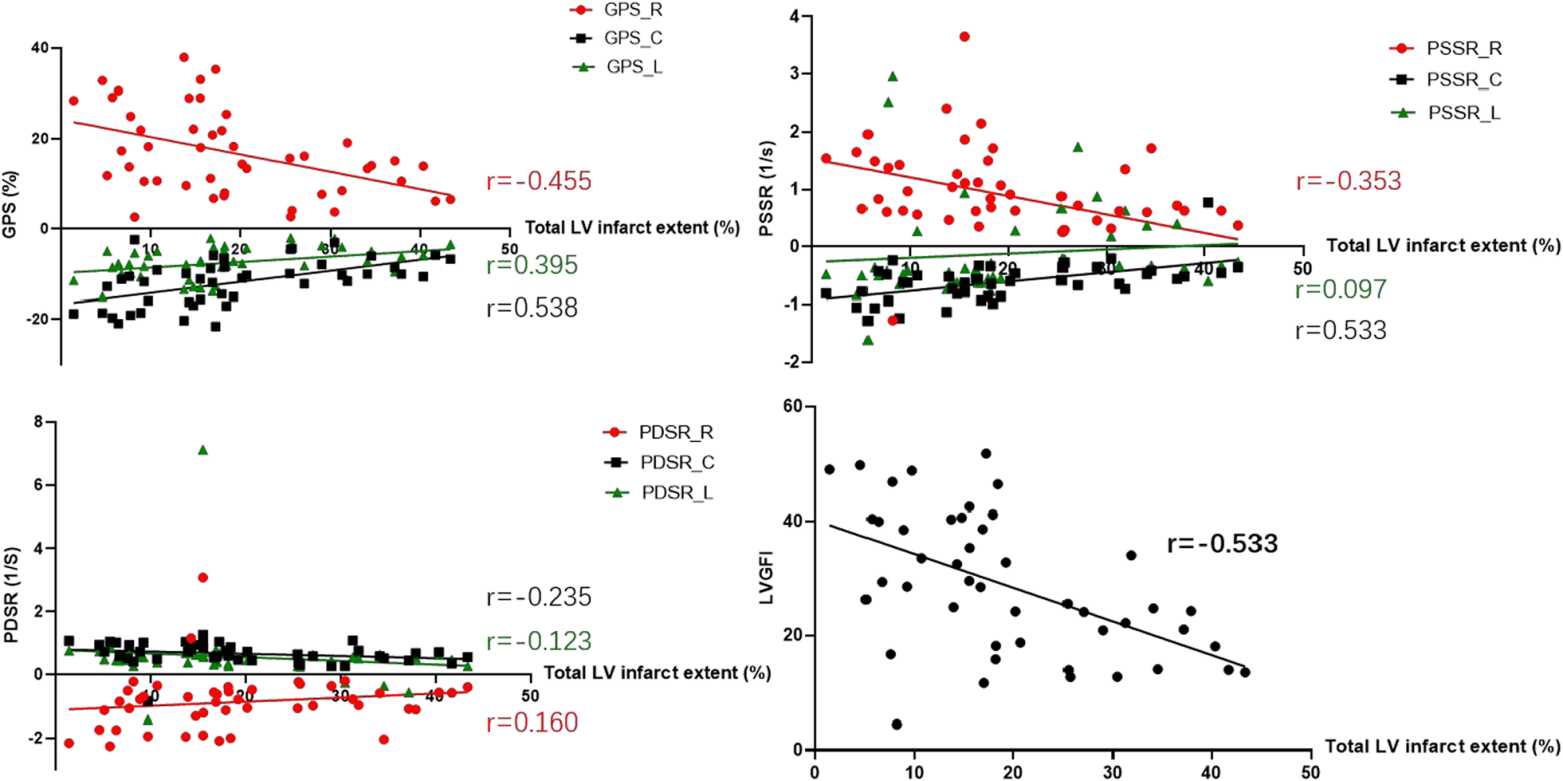
The relationship between infraction parameters and LV function, and LV deformation parameters. *GPS* global peak strain, *PSSR* peak systolic strain rate, *PDSR* peak diastolic strain rate, *R* radial, *C* circumferential, *L* longitudinal

In this study, the range of infarct size of LV and total LV infarct extent were 17.96 (11.07–25.26) and 17.12 (9.58–27.53), respectively. As shown in Table 3, for T2DM(MI+) patients, there was a negative correlation between increased infarction size and decreased LVGFI (all *p* < 0.001) and LV global PS in all three directions (all *p* < 0.01, Fig. 3). LV global radial and circumferential PSSR were inversely correlated with total LV infarct extent (r =  − 0.353, *p* = 0.016; r = 0.533, *p* < 0.001, respectively), enhanced mass of LV (r =  − 0.302, *p* = 0.042; r = 0.525, *p* < 0.001, respectively), and enhanced area of LV (r =  − 0.297, *p* = 0.045; r = 0.528, *p* < 0.001, respectively) in T2DM(MI+) patients. However, LV global longitudinal PSSR and global PDSR in the three directions showed no significant relationship with infarction size (all *p* > 0.05).

**Table 3:** Correlation analysis of LV function and global strain parameters with the myocardial infarction parameters

	Total LV infarct extent (%)		Enhanced mass (g % of LV)		Enhanced area (mL %of LV)	
	r	P	r	P	r	P
LVGFI, %	− 0.533**	< 0.001	− 0.507^**^	< 0.001	− 0.506^**^	< 0.001
PS, %						
Radial	− 0.455**	0.001	− 0.437^**^	0.002	− 0.435^**^	0.001
Circumferential	0.538**	< 0.001	0.530^**^	< 0.0001	0.533^**^	.000
Longitudinal	0.395**	0.007	0.434^**^	0.003	0.431^**^	0.007
PSSR, %						
Radial	− 0.353*	0.016	− 0.302*	0.042	− 0.297*	0.045
Circumferential	0.533**	< 0.001	0.525**	< 0.001	0.528**	< 0.001
Longitudinal	0.097	0.520	0.106	0.484	0.110	0.465
PDSR, %						
Radial	0.160	0.287	0.163	0.280	0.172	0.254
Circumferential	− 0.235	0.116	− 0.220	0.141	− 0.218	0.146
Longitudinal	− 0.123	0.417	− 0.083	0.586	− 0.071	0.639

^*^p < 0.05

^**^p < 0.01

Multivariate linear regression analysis in Table 4 demonstrated that NYHA functional class and total LV infarct extent were independently associated with global radial PS (β = − 0.400, *p* = 0.002; and β = − 0.446, *p* = 0.001, respectively; model R^2^ = 0.37) and global circumferential PS (β = 0.339, *p* = 0.006; and β = 0.530, *p* < 0.001, respectively; model R^2^ = 0.41).

**Table 4:** Univariable and multivariable linear regression analysis of LV global PS

	Radial PS				Circumferential PS					Longitudinal PS				
	Univariable		Multivariable		Univariable		Multivariable			Univariable		Multivariable		
	r	P value	r	P value	r	P value	r		P value	r	P value	r		P value
Age	− 0.107	0.480			0.057	0.707				− 0.045	0.768			
Gender	− 0.262	0.079			0.269	0.071				0.262	0.079			
NYHA functional class	− 0.400	0.006	− 0.400	0.002	0.322	0.029	0.339		0.006	0.174	0.249			
BMI	0.160	0.287			− 0.112	0.458				− 0.060	0.693			
Hyperlipidemia	0.151	0.318			− 0.200	0.182				− 0.027	0.860			
Smoking	− 0.252	0.091			0.310	0.026				0.336	0.022			
Total LV infarct extent (%)	− 0.455	0.001	− 0.446	0.001	0.538	< 0.001	0.530		< 0.001	0.395	0.007			
Anterior infarct	− 0.317	0.032			0.433	0.003				0.410	0.005	0.398		0.006

#### Association between LV deformation and LGE area in T2DM patients with MI

Regarding infarction-involved regions in the T2DM(MI+) group, 28 patients had LGE areas involving the interventricular septum, 20 involving the LV inferior wall, 13 involving the LV lateral wall, and 12 involving the LV anterior wall. No LGE areas were detected in the T2DM(MI−) group.

**Fig. 4:**
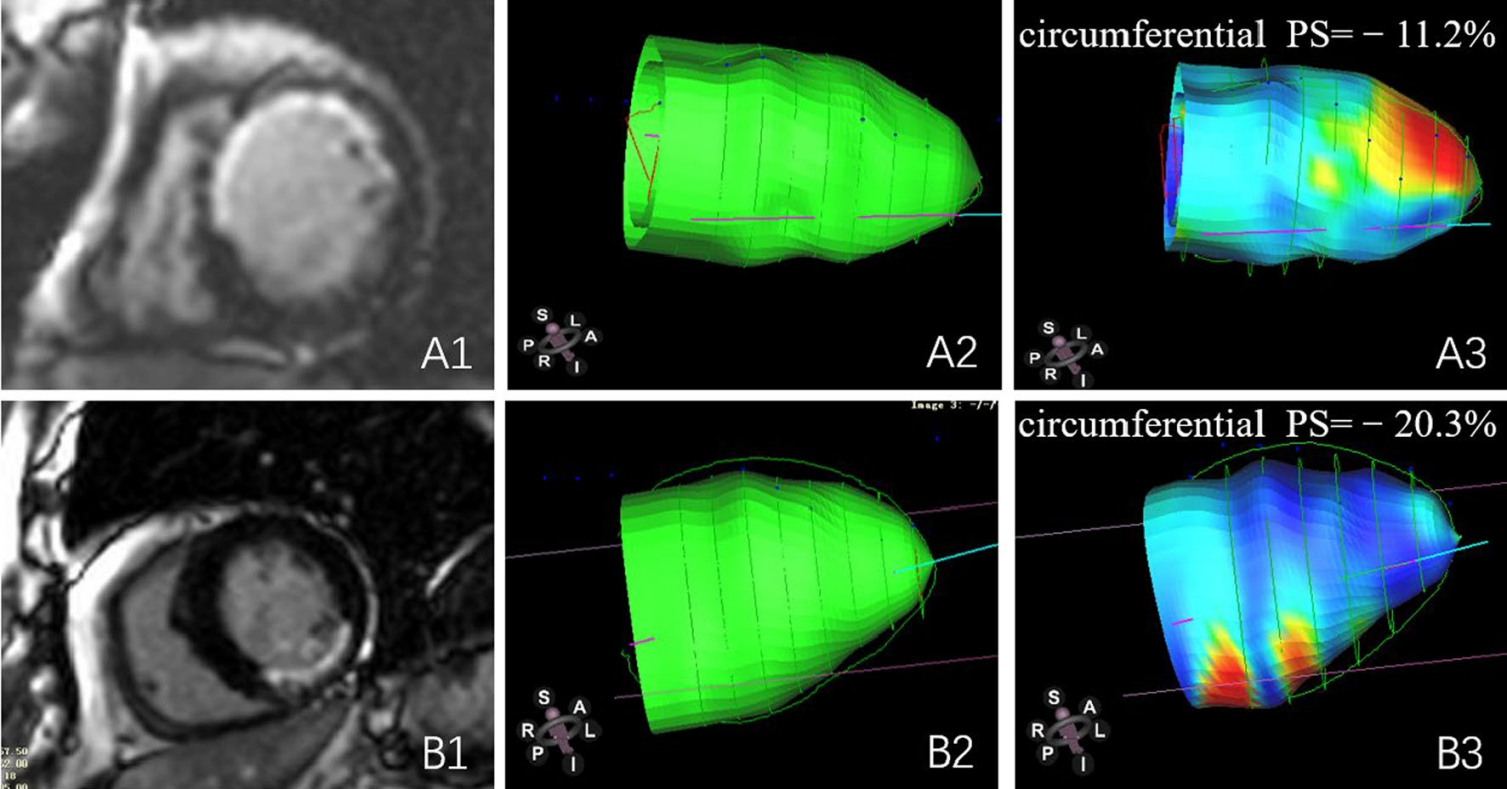
CMR-LGE images and 3D pseudo-color images of LV circumferential strain in T2DM(MI+) patients. **A1**–**3**: T2DM(MI+) patient with LV anterior wall and interventricular septum infarction, female, 58 years old, left ventricular short axis (**A1**), end-diastolic 3D pseudo-color image (**A2**), end-systolic 3D pseudo-color image (**A3**). **B1**–**3**: T2DM(MI+) patient with LV inferior wall infarction, male, 69 years old, left ventricular short axis (**B1**), end-diastolic 3D pseudo-color image (**B2**), end-systolic 3D pseudo-color image (**B3**)

**Fig. 5:**
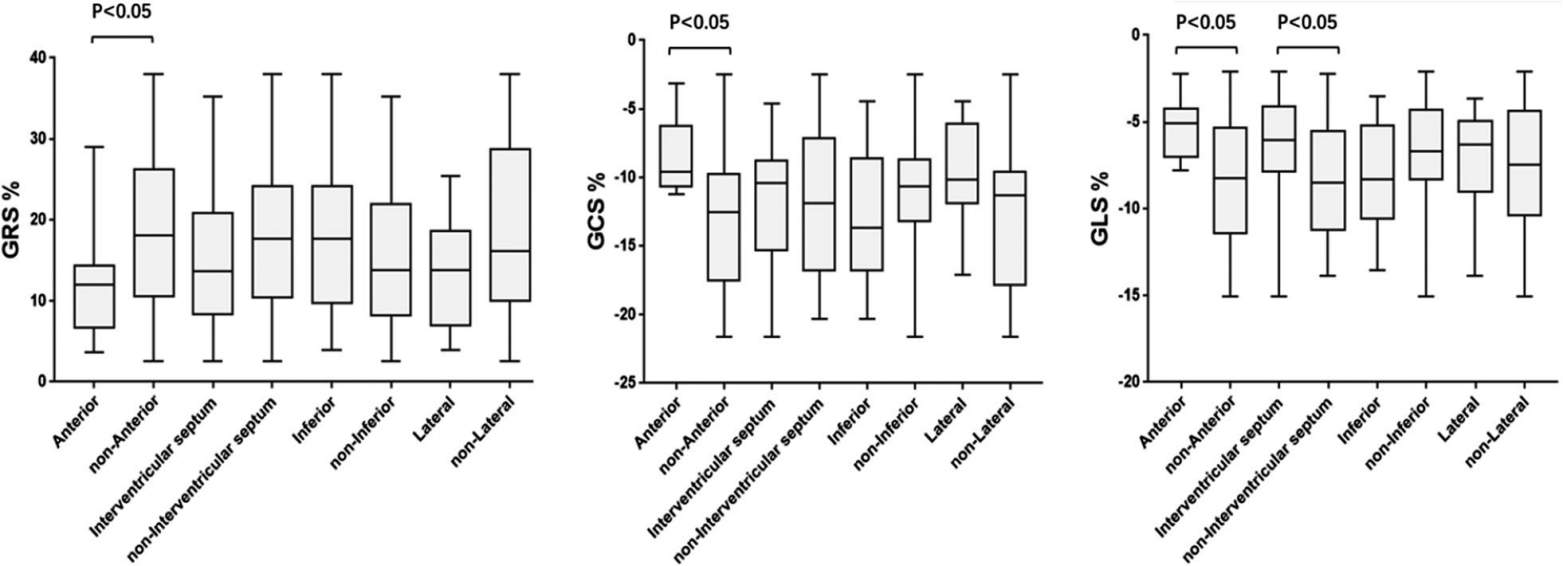
Left ventricular global PS in T2DM(MI+) patients with different area of myocardial infarct involved

For the LGE area in different regions of the LV wall, patients with anterior wall infarction had lower LV global radial PS (anterior vs. non-anterior: 11.91 ± 1.924 vs. 18.45 ± 1.671, *p* = 0.037), circumferential PS (anterior vs. non-anterior: − 8.447 ± 0.7525 vs. − 13.24 ± 0.8778, *p* = 0.034) (Fig. 4) and longitudinal PS (anterior vs. non-anterior: − 5.289 ± 0.4827 vs. − 8.351 ± 0.6052 *p* = 0.006). Patients with interventricular septum infarction have lower LV global longitudinal PS (interventricular septum vs. non- interventricular septum: − 6.515 ± 0.708 vs. − 8.503 ± 0.6702, *p* = 0.043) (Fig. 5). In addition, LV anterior wall infarction was independently associated with global longitudinal PS (β = 0.398; *p* = 0.006, model R^2^ = 0.16; Table 4).

### Inter‑ and Intraobserver variability

Table 5 summarizes the inter- and Intraobserver variabilities for LV deformation and LGE analysis. The ICCs for intra- and interobserver variabilities were 0.828–0.959 and 0.777–0.955, respectively, for LV deformation, and 0.827–0.895 and 0.882–0.893 respectively, for LGE parameters, suggesting that both techniques are in agreement.

**Table 5:** Inter- and intra-observer variability of tissue tracking and LGE

	Intra-observer (n = 70)	95% CI	Inter-observer (n = 70)	95% CI
PS, %				
Radial PS (%)	0.959	0.901–0.982	0.955	0.905–0.979
Circumferential PS (%)	0.932	0.851–0.969	0.938	0.866–0.972
Longitudinal PS (%)	0.923	0.831–0.965	0.883	0.745–0.947
PSSR, 1/s				
Radial	0.938	0.899–0.977	0.920	0.833–0.962
Circumferential	0.866	0.862–0.968	0.915	0.823–0.960
Longitudinal	0.828	0.717–0.935	0.849	0.516–0.948
PDSR, 1/s				
Radial	0.952	0.871–0.970	0.925	0.843–0.964
Circumferential	0.934	0.720–0.936	0.847	0.596–0.934
Longitudinal	0.864	0.641–0.918	0.777	0.485–0.913
LGE				
Infarct size, g	0.895	0.790–0.898	0.893	0.782–0.947
Infarct size, g % of LV	0.891	0.780–0.896	0.885	0.667–0.923
Total LV infarct extent (%)	0.827	0.544–0.905	0.882	0.651–0.893

*LGE* late gadolinium enhancement

## Discussion

T2DM is a chronic metabolic disease involving multiple organs, and the main reason for the increased mortality of patients with T2DM is DCM. In addition, T2DM is a high-risk factor for cardiovascular events such as coronary heart disease and MI [18, 19]. In this study, the following principal findings were obtained: (1) T2DM (MI−) patients have reduced LV longitudinal deformation, whereas T2DM (MI+) patients have reduced deformation in all three directions, particularly circumferential deformation; (2) in T2DM (MI+) patients, LV global PS and PSSR are decreased, related to the extent of myocardial scarring; and (3) anterior wall infarction and then ventricular septal infarction, are more likely to decrease LV deformation in T2DM (MI+) patients.

The pathogenesis of DCM is complex and multifactorial, and several causative mechanisms, including metabolic effects on myocytes, myocardial steatosis, myocardial fibrosis, microangiopathy, and autonomic nervous dysfunction [20–22]. Microvascular ischemia of DCM is most likely to occur in the sub-endocardium, where the longitudinal myocardial fibers are predominantly located; therefore, a reduction in longitudinal LV deformation occurs in the early stages of DCM. Similar to that in previous studies, our results show reduced longitudinal PS but preserved circumferential and radial PS in T2DM (MI−) patients compared to control subjects [23]. However, for T2DM(MI+) patients, deformation in all three directions of LV global PS was decreased, particularly in the circumferential direction rather than the longitudinal direction. We speculate that after MI and scar formation in T2DM patients, the myocardial cells in the scar area are replaced by the fibrous matrix, and local ventricular wall movement is reduced or impaired to varying degrees. Different from the longitudinal myocardial fibers, circumferential myocardial fibers are mainly located subepicardial, resulting in significantly reduced circumferential LV deformation [24]. Additionally, reduced longitudinal and circumferential PDSR suggests that diastolic dysfunction begins in the initial stage of DCM, which we have previously demonstrated [23] LV enlargement, particularly LVESV, and reduced LVEF, are common cardiac morphological changes after MI, which was more significant than the early diastolic functional damage of DCM. Therefore, in our study, T2DM(MI+) patients had decreased PDSR and decreased PSSR in all three directions.

LGE-CMR is considered the best available technique for noninvasive assessment of myocardial scar tissue following MI. It quantitatively evaluates the myocardial scar, discriminates the infarction transmurally, and defines the infarct territory of the LV ventricular wall [25]. The extent and size of scarring are strictly related to adverse cardiac remodeling and cardiovascular events [26]. Scar recognition is also a potential predictor for arrhythmia substrates, Histological examination has demonstrated that isolated bundles of surviving myocytes are interwoven within strands of fibrous tissue. These viable cells can form reentry circuits within fibrous tissue, thus contributing to ventricular tachycardia [27].

Previous studies have shown a correlation between the extent of MI and LV deformation in acute MI patients [9, 28] [. However, after acute MI, with the absorption of myocardial edema and inflammation, or infarction core fiber gliosis, the delayed enhancement area of LGE imaging changes. We found that on the basis of DCM, which inherently exists myocardial deformation and damage, myocardial scarring after MI has a significant negative correlation with LV radial, circumferential, and longitudinal PS and PSSR. It indicates that after acute MI, the recovery of LV myocardial systolic function and deformation is affected by the extent and quality of MI, particularly in the circumferential direction. Furthermore, we found that T2DM (MI+) patients had reduced LV global PDSR in the three directions compared with T2DM (MI−) patients, but no correlation was observed between LV global PDSR and the extent of myocardial scarring. The decrease in PDSR seems not directly associated with local myocardial scarring but may be secondary to reduced LV systolic function. Decreased LV function may further lead to myocardial metabolic disorder, and hyperglycemia may have an additive effect on diastolic dysfunction, which further leads to reduced LV PDSR. These speculations need to be verified in further studies.

At present, a few studies have explored the relationship between MI territory (involving the interventricular septum, anterior wall, lateral wall, or inferior wall) and LV global deformation and peak strain rate. The study showed that the LV global PS in all three directions is significantly affected if MI is involved in the LV anterior wall. Furthermore, anterior wall involvement is an independent factor for the decrease in LV global longitudinal PS. Most of the culprit vessels involved the infarction of the LV anterior wall was LAD, whereas the right bundle branch and left anterior fascicular are slender, and the LAD is the main blood supply. When LAD stenosis and obstruction to the blood supply might affect LV deformation. Other studies have focused on the stratification effects of the size of MI on the prognosis and survival risk and found that MI size is an important predictor of the quality of life of patients [29, 30]. However, MI involving different areas of the LV wall may result in varying LV deformation, implying that more attention should be paid to LV infarction involvement during clinical evaluation.

## Limitations

This study has several limitations. First, this study was a single-center study, so there may be some biases influencing the results. Second, the medication and NT-proBNP in the control group were not measured. However, the medical histories and the physical examination reports were carefully checked to ensure that they met the inclusion and exclusion criteria. Thirdly, as our study was a retrospective study, there are inherent design limitations, and the long-term development in T2DM patients after myocardial infarction should be further investigated in follow-up in the future. We aim to accomplish this in our future research endeavors.

## Conclusions

This study found that systolic dysfunction is more likely to occur in T2DM patients after MI, with a significant reduction in LV global circumferential deformation. The size of myocardial scarring is negatively correlated with the LV global PS and PSSR. In addition, MI in different regions has different effects on the reduction of LV deformation. Therefore, more attention should be paid to MI scarring and the extent of MI involvement in T2DM patients in clinical evaluations.

## Data Availability

The datasets used and analyzed during the current study are available from the corresponding author on reasonable request.

## Abbreviations

DM: Diabetes mellitus
T2DM: Type 2 diabetes mellitus
DCM: Diabetic cardiomyopathy
CMR: Cardiac magnetic resonance
LGE: Late gadolinium enhancement
LV: Left ventricular
LVGFI: Left ventricular global function index
MI: Myocardial infarction
PDSR: Peak diastolic strain rate
PS: Peak strain
PSSR: Peak systolic strain rate
LAD: Left anterior descending coronary artery
EDVi: End‑diastolic volume index
ESVi: End systolic volume index
SVi: Stroke volume index
LVEF: Left ventricle ejection fraction
ICC: Intraclass correlation coefficient

## References

[1] Bando YK, Murohara T (2014). Diabetes-related heart failure. Circ J.

[2] Levelt E, Gulsin G, Neubauer S, McCann GP (2018). Diabetic cardiomyopathy: pathophysiology and potential metabolic interventions state of the art review. Eur J Endocrinol.

[3] Kalam K, Otahal P, Marwick TH (2014). Prognostic implications of global LV dysfunction: a systematic review and meta-analysis of global longitudinal strain and ejection fraction. Heart.

[4] Tanaka H, Tatsumi K, Matsuzoe H, Matsumoto K, Hirata K-I (2020). Impact of diabetes mellitus on left ventricular longitudinal function of patients with non-ischemic dilated cardiomyopathy. Cardiovasc Diabetol.

[5] Chen X, Guo H, Yang Q, Fang J, Kang X (2020). Quantitative evaluation of subclinical left ventricular dysfunction in patients with type 2 diabetes mellitus by three-dimensional echocardiography. Int J Cardiovasc Imaging.

[6] Miki T, Yuda S, Kouzu H, Miura T (2013). Diabetic cardiomyopathy: pathophysiology and clinical features. Heart Fail Rev.

[7] Smith SCJ (2012). Our time: a call to save preventable death from cardiovascular disease (heart disease and stroke). Circulation.

[8] Roffi M (2016). 2015 ESC Guidelines for the management of acute coronary syndromes in patients presenting without persistent ST-segment elevation: task force for the management of acute coronary syndromes in patients presenting without persistent ST-segment elevation of. Eur Heart J.

[9] Palazzuoli A (2015). The impact of infarct size on regional and global left ventricular systolic function: a cardiac magnetic resonance imaging study. Int J Cardiovasc Imaging.

[10] Lejeune S (2021). Diabetic phenotype and prognosis of patients with heart failure and preserved ejection fraction in a real life cohort. Cardiovasc Diabetol.

[11] Bojer AS (2020). Distinct non-ischemic myocardial late gadolinium enhancement lesions in patients with type 2 diabetes. Cardiovasc Diabetol.

[12] Yan W-F (2021). Impact of type 2 diabetes mellitus on left ventricular diastolic function in patients with essential hypertension: evaluation by volume-time curve of cardiac magnetic resonance. Cardiovasc Diabetol.

[13] Kihlberg J (2020). Clinical validation of three cardiovascular magnetic resonance techniques to measure strain and torsion in patients with suspected coronary artery disease. J Cardiovasc Magn Reson Off J Soc Cardiovasc Magn Reson.

[14] Masci PG (2020). Early or deferred cardiovascular magnetic resonance after ST-segment-elevation myocardial infarction for effective risk stratification. Eur Heart J Cardiovasc Imaging.

[15] Alberti KG, Zimmet PZ (1998). Definition, diagnosis and classification of diabetes mellitus and its complications. Part 1: diagnosis and classification of diabetes mellitus provisional report of a WHO consultation. Diabetes Med..

[16] Thygesen K (2018). Fourth universal definition of myocardial infarction. J Am Coll Cardiol.

[17] Bondarenko O (2005). Standardizing the definition of hyperenhancement in the quantitative assessment of infarct size and myocardial viability using delayed contrast-enhanced CMR. J Cardiovasc Magn Reson Off J Soc Cardiovasc Magn Reson.

[18] Seferovic JP (2018). Retinopathy, neuropathy, and subsequent cardiovascular events in patients with type 2 diabetes and acute coronary syndrome in the ELIXA: the importance of disease duration. J Diabetes Res.

[19] Engelen SE (2017). Incidence of cardiovascular events and vascular interventions in patients with type 2 diabetes. Int J Cardiol.

[20] Cohen CD (2021). Diastolic dysfunction in a pre-clinical model of diabetes is associated with changes in the cardiac non-myocyte cellular composition. Cardiovasc Diabetol.

[21] Nesti L (2021). Mechanisms of reduced peak oxygen consumption in subjects with uncomplicated type 2 diabetes. Cardiovasc Diabetol.

[22] Levelt E (2016). Cardiac energetics, oxygenation, and perfusion during increased workload in patients with type 2 diabetes mellitus. Eur Heart J.

[23] Liu X (2018). Left ventricular subclinical myocardial dysfunction in uncomplicated type 2 diabetes mellitus is associated with impaired myocardial perfusion: a contrast-enhanced cardiovascular magnetic resonance study. Cardiovasc Diabetol.

[24] Mangion K, McComb C, Auger DA, Epstein FH, Berry C (2017). Magnetic resonance imaging of myocardial strain after acute st-segment-elevation myocardial infarction a systematic review. Circ Cardiovasc Imaging.

[25] Abou R (2020). Left ventricular mechanical dispersion in ischaemic cardiomyopathy: association with myocardial scar burden and prognostic implications. Eur Heart J Cardiovasc Imaging.

[26] Bulluck H, Hammond-Haley M, Weinmann S, Martinez-Macias R, Hausenloy DJ (2017). Myocardial infarct size by CMR in clinical cardioprotection studies: insights from randomized controlled trials. JACC Cardiovasc Imaging.

[27] Wong DTL (2012). Electro-mechanical characteristics of myocardial infarction border zones and ventricular arrhythmic risk: Novel insights from grid-tagged cardiac magnetic resonance imaging. Eur Radiol.

[28] Lustosa RP (2021). Changes in global left ventricular myocardial work indices and stunning detection 3 months after ST-segment elevation myocardial infarction. Am J Cardiol.

[29] Sandoval Y, Jaffe AS (2019). Type 2 myocardial infarction: JACC review topic of the week. J Am Coll Cardiol.

[30] Amier RP (2018). Long-term prognostic implications of previous silent myocardial infarction in patients presenting with acute myocardial infarction. JACC Cardiovasc Imaging.

